# Electroactive Materials Surface Charge Impacts Neuron Viability and Maturation in 2D Cultures

**DOI:** 10.1021/acsami.3c04055

**Published:** 2023-06-22

**Authors:** Teresa Marques-Almeida, Clarisse Ribeiro, Igor Irastorza, Patrícia Miranda-Azpiazu, Ignacio Torres-Alemán, Unai Silvan, Senentxu Lanceros-Méndez

**Affiliations:** † CF-UM-UP—Physics Centre of Minho and Porto Universities and LaPMET—Laboratory of Physics for Materials and Emergent Technologies, University of Minho, Braga 4710-057, Portugal; ‡ Cell Biology and Histology Department, Faculty of Medicine, Leioa 48940, Spain; § Achucarro Basque Center for Neuroscience, Leioa 48940, Spain; ∥ BCMaterials, Basque Center for Materials, Applications and Nanostructures, UPV/EHU Science Park, Leioa 48940, Spain; ⊥ Basque Foundation for Science, Ikerbasque, Bilbao 48009, Spain

**Keywords:** piezoelectric biomaterials, maturation of primary neurons, surface charge stimuli, active systems, cell culture, neural tissue

## Abstract

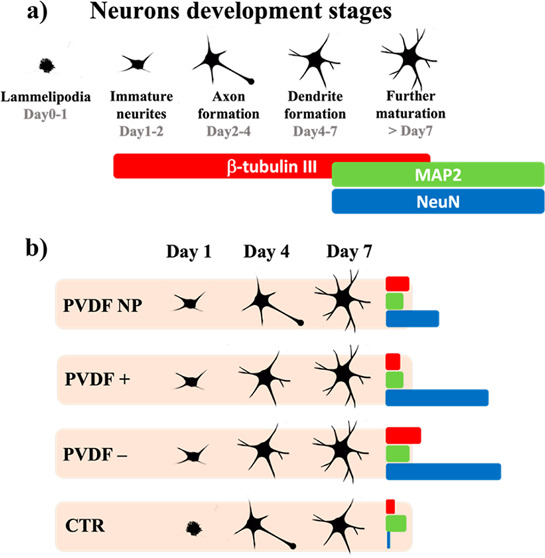

Since neurons were first cultured outside a living organism more than a century ago, a number of experimental techniques for their *in vitro* maintenance have been developed. These methods have been further adapted and refined to study specific neurobiological processes under controlled experimental conditions. Despite their limitations, the simplicity and visual accessibility of 2D cultures have enabled the study of the effects of trophic factors, adhesion molecules, and biophysical stimuli on neuron function and morphology. Nevertheless, the impact of fundamental properties of the surfaces to which neurons adhere when cultured *in vitro* has not been sufficiently considered. Here, we used an electroactive polymer with different electric poling states leading to different surface charges to evaluate the impact of the net electric surface charge on the behavior of primary neurons. Average negative and positive surface charges promote increased metabolic activity and enhance the maturation of primary neurons, demonstrating the relevance of considering the composition and electric charge of the culture surfaces. These findings further pave the way for the development of novel therapeutic strategies for the regeneration of neural tissues, particularly based on dynamic surface charge variation that can be induced in the electroactive films through mechanical solicitation.

## Introduction

Central nervous system (CNS) trauma and disease are leading causes of death and disability worldwide, emphasizing the urgent need of developing new clinical approaches for their treatment.^1,2^ As a consequence, the exploration of new strategies to promote nerve regeneration is of great importance. In this context, *in vitro* studies are very suitable for the initial analysis of the response of neural cells to external stimulation under controlled conditions. Among the methods available to analyze neuron behavior *in vitro*, 2D cultures represent a simple approach with several advantages over more complex methods.^3^

Primary neurons can be isolated from animals or humans using already established methods.^3^ Nevertheless, after extraction, *in vitro* maintenance can be quite challenging. For instance, it is difficult to culture primary neurons with morphology and behavior similar to their basic nature or even to maintain the cultures for extended periods of time in most cases due to adherence issues that are particularly prominent in case of glass coverslips and hinder imaging and electrophysiological assays.^4^

The use of electroactive materials and, in particular piezoelectric ones, for the electrical stimulation of cell cultures and tissues has been proposed.^5−7^ Among them, poly(vinylidene fluoride) (PVDF) and its co-polymers stand out due to their highest piezoelectric coefficient (d_33_) and their possibility to be processed in a large variety of tailored morphologies and microstructures.^8,9^ Depending on the processing conditions, semicrystalline PVDF can be obtained in five different crystalline phases, known as α, β, γ, ε, and δ,^8,9^ being the β-phase the most electroactive one. In addition, the dipole moments of the β-phase PVDF can be aligned by the application of an electric field. This procedure improves the piezoelectric response of the material and leads to overall net positive and net negative surfaces.^10^

PVDF substrates have been used for conventional, static cultures, where the effect of average surface charge on cell behavior is evaluated, and under dynamic cultures using mechano-electrical or magneto–electrical stimulation, which induces a variation in the surface charge and provides the cells with an electric stimulus. Since major body functions are controlled by electrical signals, PVDF scaffolds are being investigated for a variety of tissue engineering applications^5,11^ and have been shown to enhance the adhesion, proliferation, and differentiation of different cell types, including stem cells,^12^ preosteoblasts,^13−15^ myoblasts,^10,16^ and neuroblastoma^17,18^ cells. In fact, it has been proven that there is a strong influence of both electric surface charge and electrically active microenvironments on SH-SY5Y neuroblastoma cell adhesion, proliferation, and differentiation. Specifically, PVDF substrates with a net positive charge have a positive effect on the proliferation of these neuron-like cells and promote the formation of neurites.^18^ Although the cellular mechanisms involved are not well understood, a relationship between piezoelectric stimulation and activation of membrane ion channels has been proposed.^17,19^ Further and more detailed molecular studies are still needed to comprehend these interesting phenomena.

In the present work, we extend the discoveries made using neuron-like cells by analyzing the behavior of primary rat neurons when cultured for up to 2 weeks on substrates with different net charges. The main objective is to evaluate the possibility of using PVDF electrically active microenvironments to improve neuron growth cultures and eliminate the major limitations associated with conventional 2D culture settings. For that reason, PVDF, the polymer with the highest dielectric and electroactive responses, was selected instead of other electroactive biopolymers, with lower polarity, electroactivity, and signal stability over time.^18^ Our results reveal that non-transformed neural cells display increased viability at early timepoints when seeded on PVDF substrates with a net charge, either positive or negative. Furthermore, the expression of maturation markers such as NrCAM, N-Cad, and NeuN is also upregulated in neurons cultured on negative surfaces. Taken together, our work highlights the importance of considering the electric charge of the culture surface when working with cells of the nervous system, demonstrating the suitability of 2D substrates based on surface-charged materials and, in particular, of PVDF due to its outstanding electroactive characteristics.^18^

## Materials and Methods

β-Phase PVDF films with a thickness of 110 μm were obtained through the solvent casting method, according to refs (20), and (21). Poled films with average positive (PVDF+) and negative (PVDF−) surface charges, with a d_33_ response of −24 pC/N measured by a d_33_ meter (model 8000, APC Int Ltd), and non-poled films (PVDF NP) with average zero net charge were produced. The average surface potential of the different samples is thus 6 V for PVDF+, −4 V for PVDF–, and 0 V for PVDF NP, respectively (Table 1). The characterization of β-phase PVDF films is already well-established in the literature, with no appreciable differences between the different processed films in terms of β-phase content, crystallinity degree, or surface roughness (Table 1). The surface contact angle can be affected by the poling process, which leads to an increase in material surface energy and consequently to lower contact angles for non-poled samples; however, all the samples present a hydrophilic behavior.^16^ Therefore, any behavioral change in neurons between the different studied PVDF films will result from the net surface charge effect.

**Table 1:** Relevant Characteristics of β-Phase PVDF Films

	PVDF NP	PVDF+	PVDF–
average surface charge	neutral (0 V)^22^	positive (6 V)^22^	negative (−4 V)^22^
β-phase content (%)	≈80^23^		
crystallinity degree (%)	≈46^24^		
contact angle (°)	≈83^16^	≈51^16^	≈45^16^
surface roughness (nm)	≈42^12^		

Glass coverslips of 12 mm diameter (Fisher) were used as control during all the experiments. 13 mm diameter PVDF discs and coverslips were placed in 24-well plates, sterilized under ultraviolet (UV) light for 30 min on both sides, washed twice with phosphate buffer saline (PBS 1x), and coated with poly-l-lysine (PLL, Sigma-Aldrich). For coating, samples were covered with a gelatin solution (0.25 mg/mL, Sigma-Aldrich) for 30 min and with a PLL solution (0.1 mg/mL in borate buffer 0.15 M, pH 8.4) for 1 h and 30 min, respectively. After being rinsed twice with PBS and once with Mili-Q water, all materials were exposed to UV for 2 h 30 and incubated at 37 °C until use. Samples were covered for 30 min with 10% fetal bovine serum (FBS, Thermofisher) in PBS, right before use.

### Cortical Neuronal Cultures

For primary neuron isolation, postnatal P0–P3 rats were used. The rats were decapitated, and their brains were extracted and placed in a sterile Petri dish with ice-cold Earle’s balanced salt solution buffer (EBSS, Fisher). Under a microscope, cortical hemispheres were dissected, their meninges removed, collected in a sterile Petri dish, and mechanically fragmented with a sterile razor blade. To isolate primary neurons, tissue fragments were enzymatically digested for 1 h inside an incubator at 37 °C and 5% CO_2_ in Papain Medium composed of EBSS, 2.5 U/mL papain enzyme (Worthington), 5 mM papain activator (5 mM l-cysteine, 2 mM EDTA, 0.067 mM β-mercaptoethanol), 100 U/mL DNAse (Sigma), and 2 mM calcium–magnesium (Sigma). The resulting suspension was vortexed for 2 min and left for 5 min to settle, and the resultant supernatant was collected and centrifuged (1100 rpm, 5 min). Papain inhibitor medium [1:10 papain inhibitor (Worthington) in EBSS buffer] was added to the cell pellet and centrifuged (1050 rpm, 5 min). The resulting cell pellet was resuspended in Neurobasal medium (Gibco), and a cell suspension with 1.4 × 10^5^ cells/mL was seeded on each substrate.

### Cell Viability Assay and Optical Microscopy Imaging

After 7 days of culture, viable cells were measured using a CellTiter 96 AQueous One Solution Cell Proliferation Assay (MTS, Promega). For that, cells were incubated in an MTS-neurobasal medium (1:5) solution, for 3 h at 37 °C, 5% CO_2_ and 95% humidified air atmosphere. The absorbance was measured at 490 nm with a microplate reader, and the obtained results are shown as the mean ± standard deviation (SD) of triplicate samples. Statistical analysis was performed using the GraphPad Prism program with one-way ANOVA. Cells were monitored with an optical microscope (Olympus CKX53), and pictures were taken at day 1, 4, and 7 to evaluate their behavior on top of each material.

### Immunofluorescence

For immunofluorescence visualization, cells were fixed with methanol (Sigma-Aldrich) for 6 min at −20 °C and blocked for 1 h with a blocking solution (1% Triton X-100 and 2.5% BSA in PBS) at room temperature (RT). After blocking, Anti-Tubulin β-III primary antibody (Merck) (1:100 in blocking solution) was added to neurons and incubated overnight at 4 °C, followed by incubation with Donkey anti-Rabbit IgG (H + L) Highly Cross-Adsorbed Secondary Antibody, Alexa Fluor 488 (Thermofisher) for 1 h at RT in the dark. Nuclei were stained with 4′,6-diamidino-2-phenylindole (DAPI, Fisher, 1:1000 in PBS), 15 min at RT in the dark. Samples were washed 3 times with PBS between steps. After sample mounting with Fluoroshield (Sigma-Aldrich), fluorescence images were obtained with a high-content screening system (HCS, Cellinsight CX7, Thermo Scientific) and a confocal microscope (Leica SP8). All images were acquired with the same settings and quantified using ImageJ software. For that, 50 images were selected, and their signal intensity was measured using the HCS software. Results are presented as mean ± SD, and statistical analysis was performed with GraphPad Prism using two-way ANOVA. At day 7 and day 14, nuclei per image area were counted with ImageJ software to determine cell viability. For that, 20 images obtained by HCS were analyzed; results are presented as mean ± SD, and statistical analysis was performed as above.

### Scanning Electron Microscopy Imaging

Cells were fixed in 2% glutaraldehyde (Sigma-Aldrich) for 30 min at RT, washed with PBS, and dehydrated in a graded series of ethanol for 20 min each. After that, hexamethyldisilazane (Sigma-Aldrich) was added to the samples and incubated at RT for 20 min. After drying, samples were glued onto aluminum pin stubs with electrically conductive carbon adhesive tape (Electron Microscopy Sciences). A thin layer of gold was sputtered on the materials surface by magnetron sputtering (Polaron, model SC502) and evaluated with a Hitachi-S4800 scanning electron microscope with a 5 kV beam acceleration.

### Quantitative Polymerase Chain Reaction

For gene expression analysis, total RNA was extracted from each sample using the PureLink RNA Mini Kit from Ambion (Life Technologies), according to the manufacturer’s instructions. Briefly, cells were lysed with Lysis Buffer with 2-mercaptoethanol and homogenized using a 21-gauge syringe needle. RNAs were washed 3 times, eluted from the spin cartridge with RNAse-free water and stored at −20 °C.

Quantitative polymerase chain reaction (qPCR) was performed in a real-time PCR system (BioRad). The results are presented as relative gene expression compared with the housekeeping gene actin using the 2^–ΔΔCt^ method,^25^ and statistical analysis was performed using GraphPad Prism with one-way ANOVA. PCR primer sequences used are described in Table 2.

**Table 2:** qPCR Primers

gene	forward/reverse	Primer	size (bp)	melting temperature (*T*_m_) (°C)
**N-cadherin**	forward	5′-ACTGAGGAGCCGATGAAGGAACCAC	258	66.29
	reverse	5′-GTTGATGATGAAGATGCCCGTTGGA		63.61
**NrCAM**	forward	5′-AACAACTGTGGACGAAGCTG	192	58.70
	reverse	5′-CATTCTTCTTTGCTGCCTGC		58.01
Β**-IIItubulin**	forward	5′-TGAGGCCTCCTCTCACAAGT	237	60.18
	reverse	5′-TGCAGGCAGTCACAATTCTC		58.47
**MAP2**	forward	5′-CAGCTGCACTGGAAGAAGC	77	59.13
	reverse	5′-TAAAGGCTCAGCGAATGAGG		57.8
**NeuN**	forward	5′-TATGGTGCTGAGATTTATGGAG	133	55.40
	reverse	5′-CGATGGTATGATGGTAGGGAT		56.45

## Results and Discussion

Primary neurons were isolated from neonatal (P0–P3) rats and immediately seeded on positively poled PVDF (PVDF+), negatively poled PVDF (PVDF−), non-poled PVDF (PVDF NP) surfaces, and on glass coverslips (CTR). We then examined the morphological and functional variations among the cells cultured on the different surfaces. Given that all PVDF variants have a similar chemical composition and degree of surface roughness (Table 1) and that surface charge and charge density affect the material’s wettability, the effect of the various surfaces on neuron behavior can be directly related to the net charge of those surfaces.

### Adhesion and Neurite Outgrowth Are Promoted by Poled PVDF Substrates

**Figure 1:**
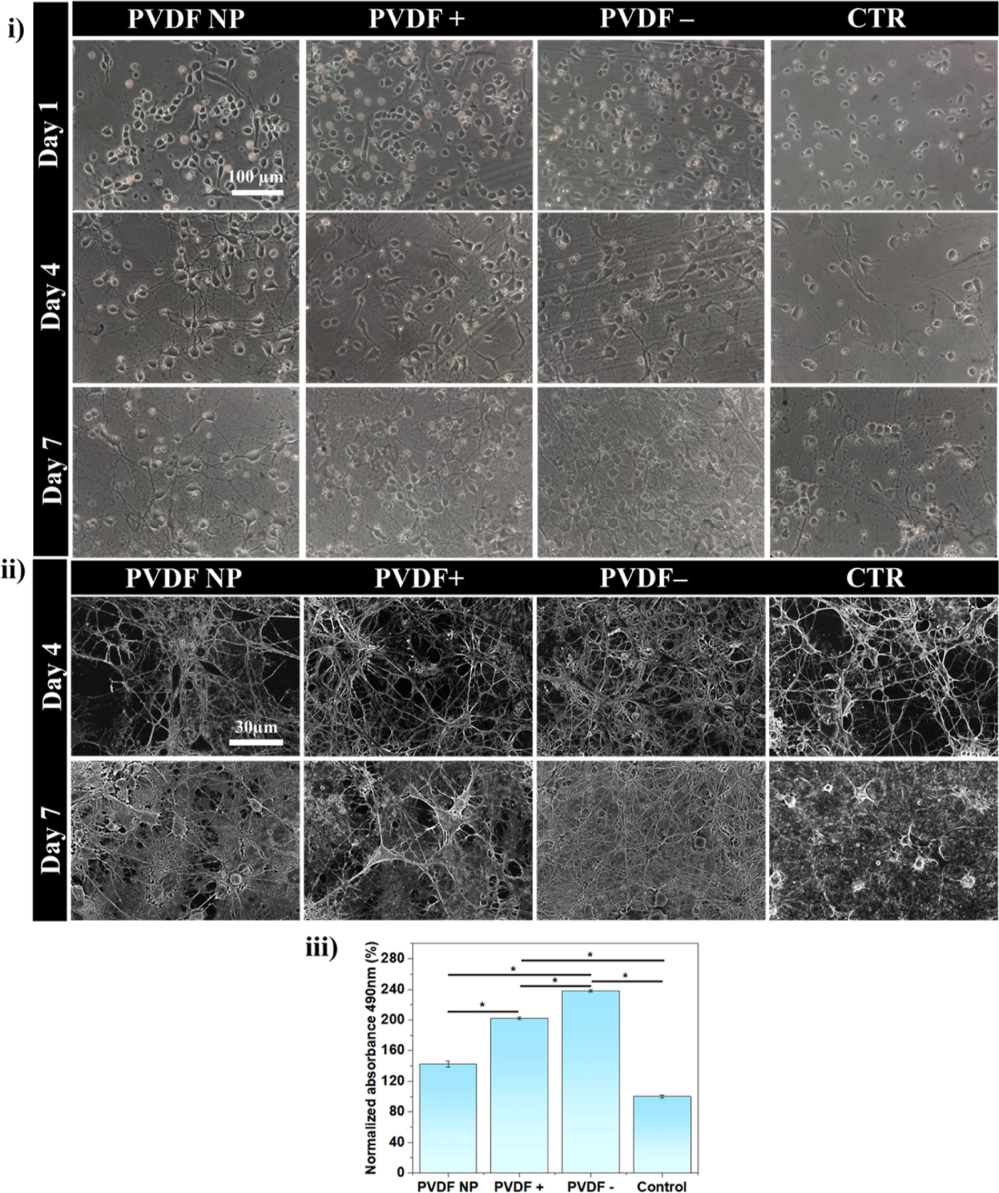
(i) Optical microscopy images of neurons cultured for 1, 4, and 7 days on different surfaces (representative scale bar of 100 μm). (ii) SEM micrographs of primary neurons cultured for 4 and 7 days (representative scale bar of 30 μm). (iii) Metabolic activity assessed with an MTS assay of primary neurons after 7 days of culture on PVDF and control substrates with one-way ANOVA statistics: *p < 0.0001.

Cultured neurons were evaluated throughout the assay using phase-contrast microscopy, and images were acquired at days 1, 4, and 7 (Figure 1i). These images show more neurons per unit area on PVDF substrates at day 1 than in standard culture surfaces (control—CTR). This suggests that this polymer promotes primary neuron attachment. Moreover, neurons present higher development of neuronal processes, *i.e.*, axons and dendrites, during the first 24 h on all PVDF substrates, independently of their surface charge. At day 7, neurons cultured on PVDF+ and PVDF– substrates seem to present more branched neurite arborization than on non-poled substrates (PVDF NP and CTR). These results suggest that the presence of surface charges, particularly negative ones, may lead to a higher level of neuron maturation.

SEM micrographs enable for more in-depth study of the neurons grown on various substrates as well as the observation of neuronal processes such as axons and dendrites, which are maturation indicators of primary neurons (Figure 1ii). It can be observed that at day 4, neurons display a more mature phenotype when cultured on PVDF poled films than on conventional culture substrates (CTR) or non-poled PVDF (PVDF NP), presenting a higher number of neurite processes that cover almost the entire surface of the materials, particularly on PVDF–. These findings indicate that early maturation is increased in cells adhering to poled surfaces. At day 7, the tendency to higher levels of maturation is maintained for all PVDF films but is particularly evident in cultures on PVDF–. Between days 4 and 7, the neuron processes increase significantly in cells cultured on PVDF NP, a phenomenon that is not evidenced for CTR conventional culture. These findings indicate that the choice of the PVDF charge can be used to tune neurons attachment and processes development.

The MTS assay is a colorimetric method to assess metabolic activity based on the reduction of the MTS-tetrazolium salt into formazan by specific enzymes. This way, the assay outcome depends on several variables, including cell number, mitochondrial activity, and cell metabolism.^26^ In our experiments, the colorimetric signal was significantly higher for cultures on PVDF films, poled and non-poled, compared to CTR (Figure 1iii). Regarding the surface charge effect, MTS reduction was significantly higher in cells cultured on charged surfaces, especially on negative ones (PVDF−), displaying a signal 3 times higher than in control cultures and around 1.7 times higher than in the absence of charge (PVDF NP). Comparing this data with the microscopy images (Figure 1i) and assuming that primary neurons are non-proliferative cells, this higher colorimetric signal can be related either by higher adherence to the substrates, leading to higher survival levels or by increased metabolic activity, related to the maturation processes.

### Neuron Maturation Is Promoted by Poled PVDF Substrates

**Figure 2:**
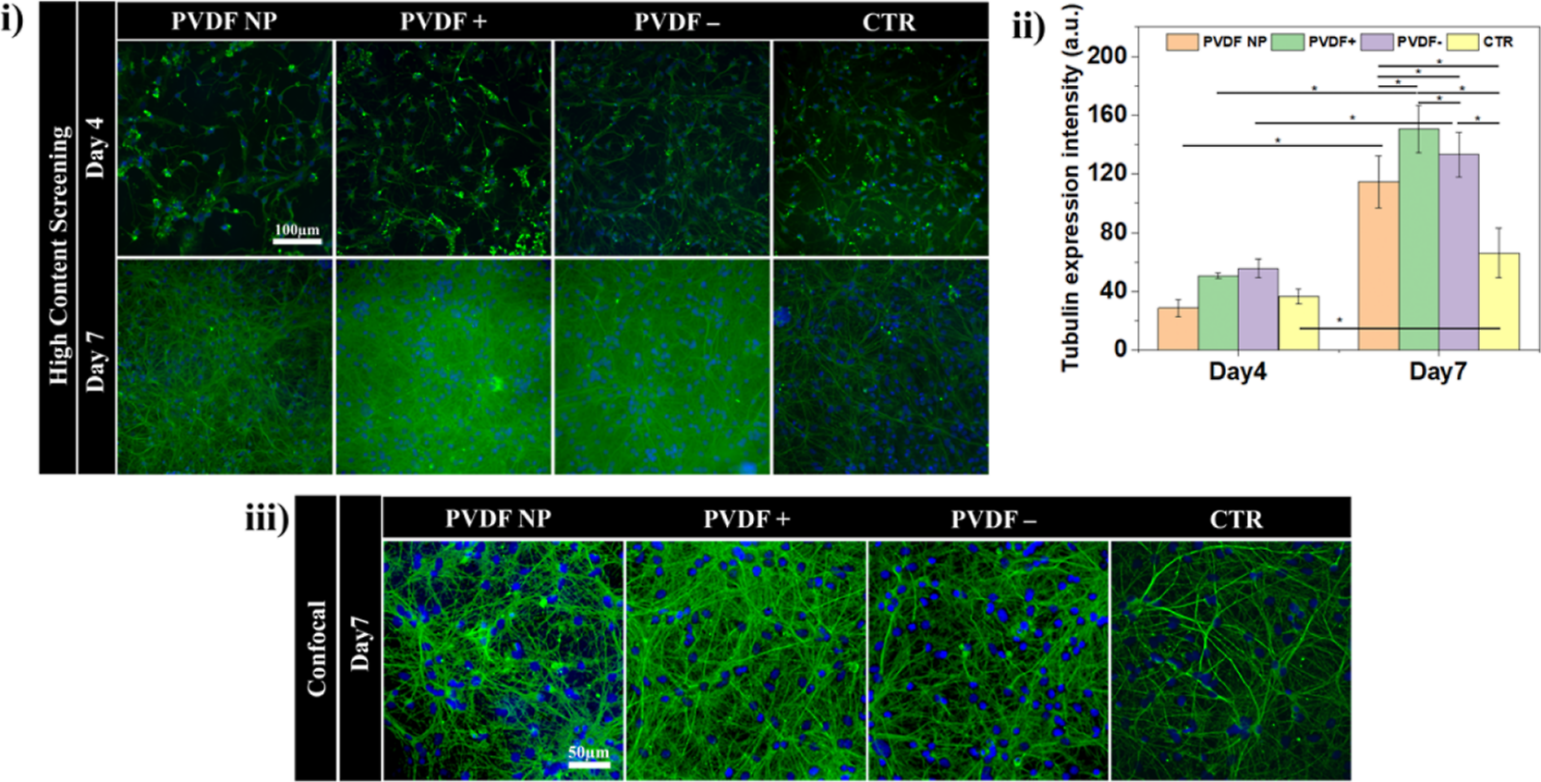
(i) HCS images of β-III tubulin (green) and nucleus (DAPI blue) at days 4 and 7, with a representative scale bar of 100 μm. (ii) Mean β-III tubulin signal intensity with two-way ANOVA statistics: *p < 0.0001. (iii) Confocal microscopy images of β-III tubulin (green) and nucleus (DAPI blue) after 7 days of culture (representative scale bar of 50 μm).

To further analyze the maturation of neurons adhering to PVDF surfaces, we stained the cultures with the neuronal marker β-III tubulin and acquired images using a HCS microscopy system at days 4 and 7 (Figure 2i). The fluorescence intensity was measured in 50 images for each condition and is presented as mean ± SD in Figure 2ii. β-III Tubulin is a structural component of the cytoskeleton of neurons, and its expression correlates with early stages of neuronal differentiation. It is classically used as a marker of the development of neuronal processes. All cells present β-III tubulin signal, which increases significantly from day 4 to day 7 for all culture conditions (Figure 2ii). This increment after 7 days is larger for neurons cultured on all types of PVDF substrates compared to the control. In turn, comparing neuron cultures on surfaces with different polarizations, poled surfaces exhibit significantly higher tubulin expression than non-poled ones at day 4, a tendency that is maintained at day 7.

We further acquired high-resolution images using confocal microscopy at day 7 of culture (Figure 2iii). These images display a clear difference in neurite outgrowth and tubulin intensity between poled and non-poled surfaces, with PVDF+ and PVDF– presenting a higher density of neuronal processes and tubulin intensity than PVDF NP and CTR.

### Primary Neurons Are Maintained for 14 days on PVDF Substrates

**Figure 3:**
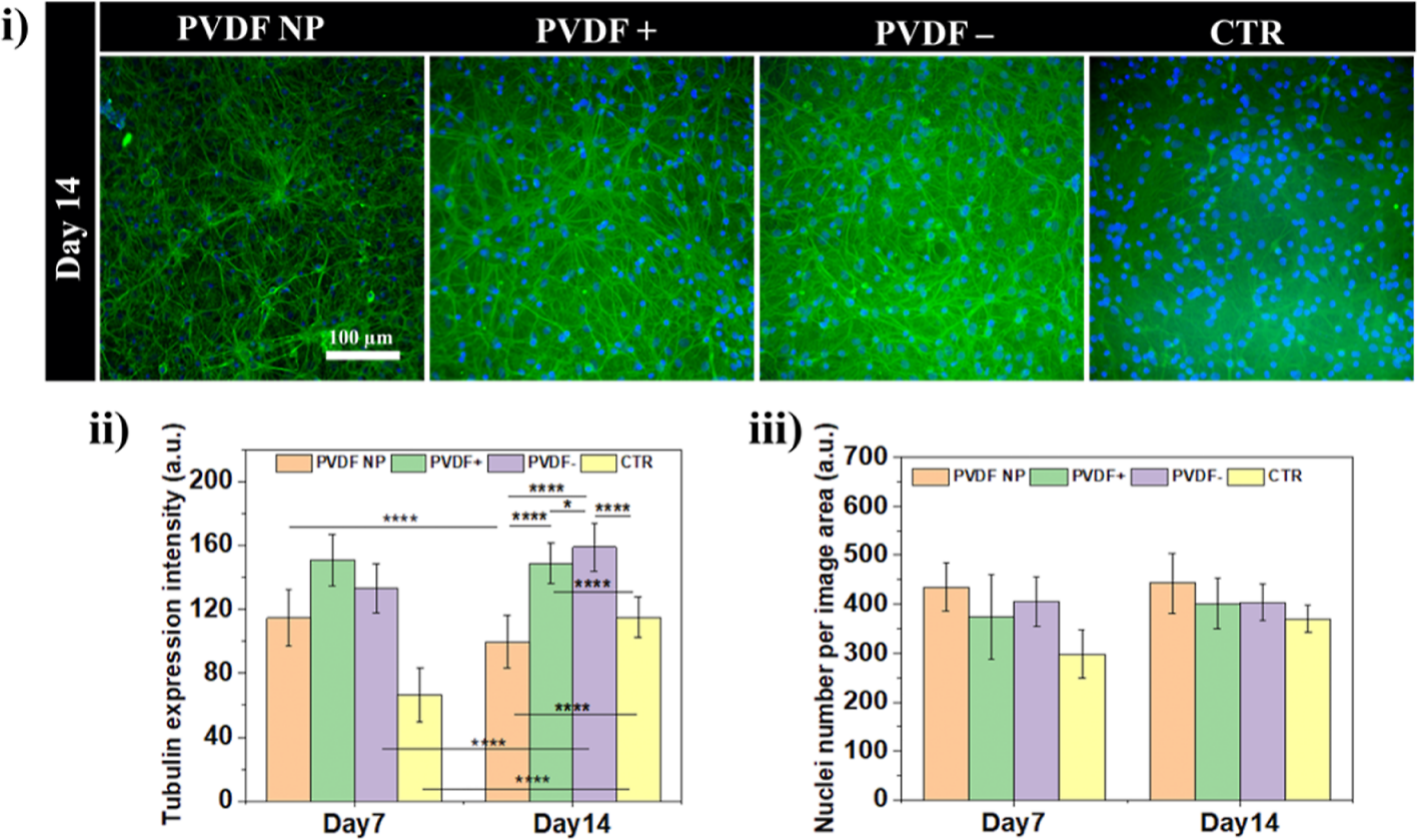
(i) HCS imaging of β-III tubulin (green) and nucleus (DAPI blue) in neurons cultured for 14 days (representative scale bar of 100 μm). (ii) Mean tubulin expression intensity at day 7 (repeated for comparison) and day 14, with two-way ANOVA statistics: *p < 0.05, ****p < 0.001. (iii) Mean nuclei number per image for all conditions at day 7 and day 14.

Neurons were maintained in culture for additional 7 days, making a total of 2 weeks, to examine their long-term behavior on the different substrates. At day 14, the distribution and intensity of β-III tubulin labeling (Figure 3i,ii) were analyzed, and the cell density was quantified (Figure 3iii).

After 14 days in culture, neurons show a strong tubulin staining (Figure 3i), with neurons on PVDF– and in control conditions displaying a stronger signal than after 7 days culture. In turn, neurons on PVDF NP displayed a lower signal intensity compared to day 7, and neurons cultured on PVDF+ had a nearly identical signal intensity at days 7 and 14 (Figure 3ii). Although CTR presents a higher signal of the β-III tubulin staining at day 14 than at day 7, those levels are still lower than any of the values obtained for PVDF at day 7 and also lower than for poled PVDF at day 14. The number of cells remained constant between days 7 and 14, according to the nuclei count (Figure 3iii). These results suggest that neurons reach their initial maturation stage much faster (around day 7) when cultured on PVDF surfaces and likely start expressing factors related to late differentiation stages. To test this hypothesis, we analyzed the expression of a number of genetic markers in neurons cultured for 7 days.

### Negatively Charged PVDF Upregulates Neuronal Marker Expression

Gene expression of different neuronal markers was evaluated at day 7 using qPCR and the primers listed in Table 2.

**Figure 4:**
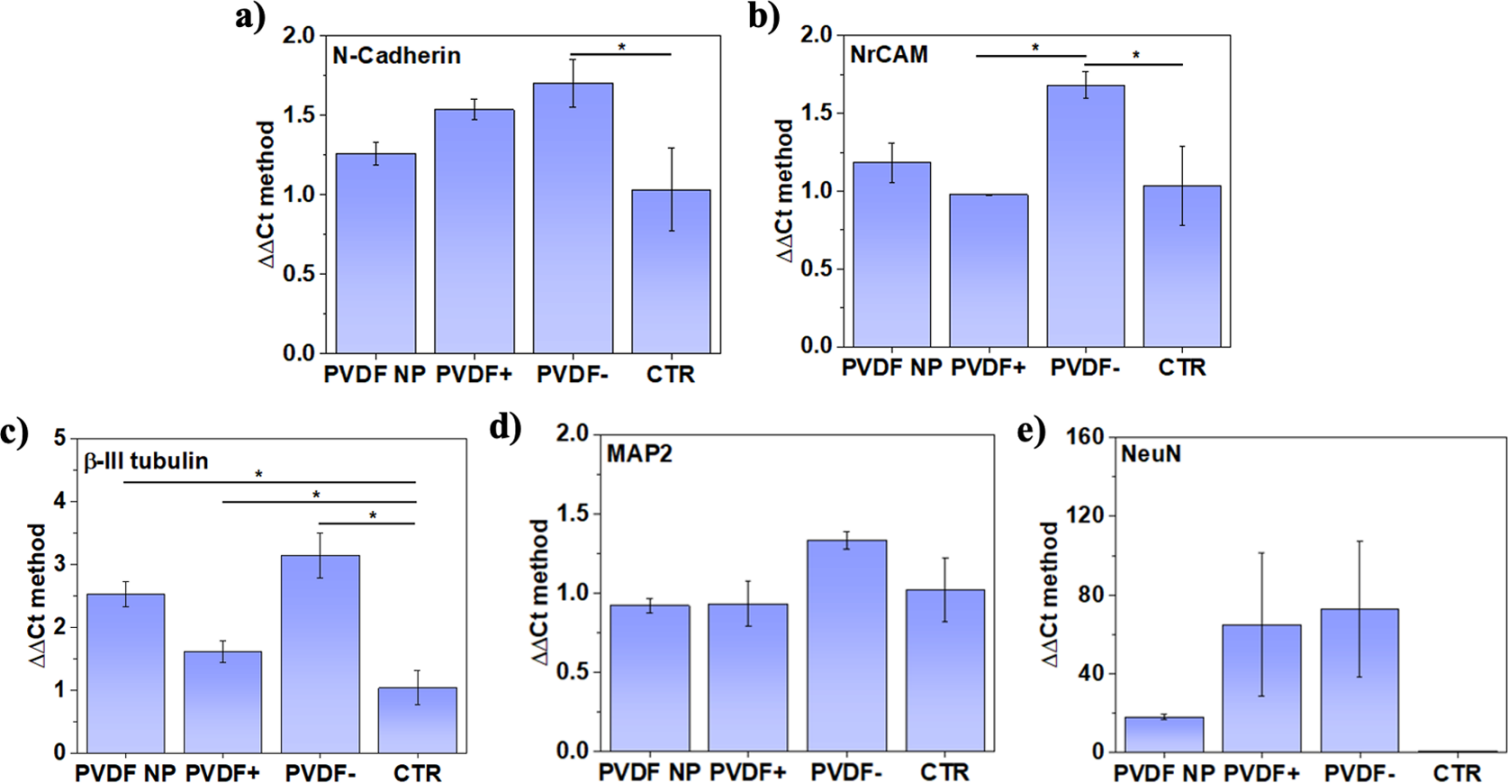
Gene expression of neuronal markers after 7 days of culture. (a) N-Cadherin, (b) NrCAM, (c) β-III tubulin, (d) MAP2, and (e) NeuN. Statistical analysis with one-way ANOVA: *p < 0.05.

**Figure 5:**
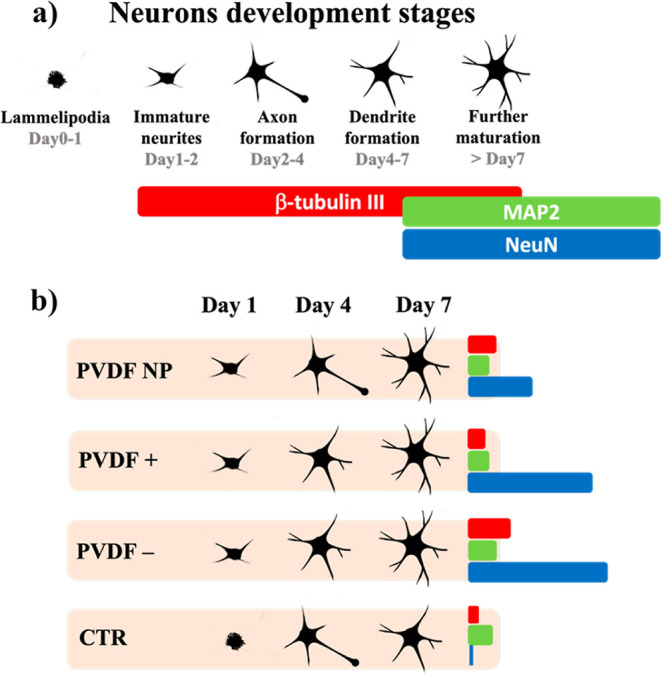
(a) Neuron maturation over time and associated markers. (b) Schematic representation of the response of neurons to the different culture conditions.

The establishment of neuron–neuron connections is necessary for the correct development of neural circuits. These contacts are mediated by cell adhesion molecules such as neural-cadherin (N-cadherin or Cadherin-2) and neuronal cell adhesion molecule (NrCAM). Specifically, N-cadherin plays a central role in the early stages of synaptogenesis, dendritic arborization, axon guidance, and neurite outgrowth.^27,28^ In turn, NrCAM is involved in neurite outgrowth, being present in many cellular processes, including axonal pathfinding and myelination, cell migration, and fasciculation of nerve fibers [14]. Since neurons exhibit more cellular processes when adhering to PVDF surfaces, we examined the expression of these adhesion molecules and found a statistically significant increase in their expression in neurons cultured on PVDF– (Figure 4a,b). We further analyzed the gene expression of β-III tubulin, which is an early-stage differentiation marker, and MAP2 and NeuN, that are characteristic of mature neurons. As expected, β-III tubulin gene expression was significantly higher for all PVDF samples comparing to the control (CTR) (Figure 4c). In turn, although not statistically significant, an increase in the expression of the mature neuron markers MAP2 and NeuN in cells cultured on PVDF– was observed (Figure 4d,e). These findings confirm that, in contrast to standard culture conditions, negatively charged PVDF promotes early neurite outgrowth and neuronal development (Figure 5).

## Conclusions

In the present work, we have analyzed the response of primary neurons when cultured on PVDF surfaces with different surface electric charges. Since the PVDF used in our experiments has otherwise identical surface properties, the effect of the various surfaces on neuron behavior can be directly related to the net charge of those surfaces. Our findings show that PVDF films promote the attachment, viability, and maturation of primary neurons. More cells per unit area and longer neurites were already present in neurons cultured for one day on PVDF surfaces. Early neurite outgrowth was induced using poled PVDF as culture substrates, since by day 4 of culture, neurons already showed significantly increased neural process development, and by day 7, this development was even more obvious in neurons cultured on PVDF–. Negative surface charges particularly increased cell metabolism, being about 3 times higher than in control and around 1.7 times higher than in the absence of charge (PVDF NP). When adhering to PVDF–, neurons undergo maturation at a faster rate, as revealed by the higher expression levels of MAP2 and NeuN. Taken together, our results highlight the importance of considering the composition and electric charge of the culture surface for the *in vitro* maintenance of neurons, demonstrating the suitability of 2D PVDF-based surface-charged substrates.
